# Supplementary material for: Barriers and facilitators of provision of telemedicine in Nigeria: A systematic review
Source: PLOS Digit Health. 2025 Jul 10;4(7):e0000934. doi: 10.1371/journal.pdig.0000934 (PMC12244542; doi:10.1371/journal.pdig.0000934)
Supplement: S1 Appendix — (DOCX) [file pdig.0000934.s001.docx]

**S1 Appendix**. **Sample of Search Strategy conducted for the systematic review of barriers and facilitators of telemedicine in Nigeria across PubMed, CINAHL and Scopus databases.**

| **Serial** | **Search Word** |
| --- | --- |
|  | **PubMed** |
| 1. | “Telemedicine” [Tiab] |
| 2. | “Telepharmacy” [Tiab] |
| 3. | “eHealth” [Tiab] |
| 4. | “mHealth” [Tiab] |
| 5. | “Mobile health” [Tiab] |
| 6. | “Electronic medical system” [Tiab] |
| 7. | “EMR” [Tiab] |
| 8. | 1 or 2 or 3 or 4 or 5 or 6 or 7 |
| 9. | “Nigeria” [Tiab] |
| 10. | 8 AND 9 |
|  | **CINAHL** |
| 11. | “Telemedicine” [Tiab] |
| 12. | “Telepharmacy” [Tiab] |
| 13. | “eHealth” [Tiab] |
| 14. | “mHealth” [Tiab] |
| 15. | “Mobile health” [Tiab] |
| 16. | “Electronic medical system” [Tiab] |
| 17. | “EMR” [Tiab] |
| 18. | 11 or 12 or 13 or 14 or 15 or 16 or 17 |
| 19. | “Nigeria” [Tiab] |
| 20. | 18 AND 19 |
|  | **Scopus** |
| 21. | “Telemedicine” [Tiab] |
| 22. | “Telepharmacy” [Tiab] |
| 23. | “eHealth” [Tiab] |
| 24. | “mHealth” [Tiab] |
| 25. | “Mobile health” [Tiab] |
| 26. | “Electronic medical system” [Tiab] |
| 27. | “EMR” [Tiab] |
| 28. | 21 or 22 or 23 or 24 or 25 or 26 or 27 |
| 29. | “Nigeria” [Tiab] |
| 30. | 28 AND 29 |
