# Supplementary material for: Barriers and facilitators of provision of telemedicine in Nigeria: A systematic review
Source: PLOS Digit Health. 2025 Jul 10;4(7):e0000934. doi: 10.1371/journal.pdig.0000934 (PMC12244542; doi:10.1371/journal.pdig.0000934)
Supplement: S2 Appendix — (DOCX) [file pdig.0000934.s002.docx]

**S2 Appendix.** **Quality Appraisal of Qualitative Studies using the Joanna Briggs Institute Criteria for Qualitative Research.**

| Study | Q1 | Q2 | Q3 | Q4 | Q5 | Q6 | Q7 | Q8 | Q9 | Q10 | Risk of Bias |
| --- | --- | --- | --- | --- | --- | --- | --- | --- | --- | --- | --- |
| Eze and Okojie 2016 | Y | Y | Y | Y | Y | ? | Y | Y | Y | N | H |
| Ebenso et al 2021 | Y | Y | Y | Y | Y | ? | Y | Y | Y | Y | H |
| van Gurp et al 2015 | Y | Y | Y | Y | Y | ? | ? | Y | Y | Y | H |
| Shekoni et al 2024 | Y | Y | Y | Y | Y | ? | Y | N* | Y | Y | H |
| Itanyi et al 2023 | Y | Y | Y | Y | Y | ? | N | N* | Y | Y | L |
| Obi-Jeff et al 2022 | Y | Y | Y | Y | Y | ? | Y | N* | Y | Y | L |
| Obi-Jeff et al 2021 | Y | Y | Y | Y | Y | ? | Y | N* | Y | Y | L |

Key: Y: Yes, N: No, L: Low, H: High. Q1: Congruity between philosophical perspective and research methodology; Q2: Congruity between research methodology and objective; Q3: Congruity between research methodology and data collection methods; Q4: Congruity between research methodology and representativeness and interpretation; Q5: Congruity between research methodology and interpretation Way; Q6: Locating researcher theoretically or culturally; Q7: No influence of researcher on research; Q8: Representation of participants and voice; Q9: Ethical approval from appropriate authority; Q10: Relationship between conclusion to analysis or data interpretation; *: small sample size; ?: unclear.
